# Specific Early Childhood Experiences Predict Executive Function Skills During Later Childhood and Adolescence: Evidence from the ECHO Cohort

**DOI:** 10.3390/ijerph23070904

**Published:** 2026-07-15

**Authors:** Colin Drexler, Maxwell Mansolf, Destany Calma-Birling, Phillip Sherlock, Courtney K. Blackwell, Philip David Zelazo

**Affiliations:** 1 Institute of Child Development, University of Minnesota, Minneapolis, MN 55455, USA; 2 Department of Medical Social Sciences, Northwestern University, Chicago, IL 60611, USA; maxwell.mansolf@northwestern.edu (M.M.); ckblackwell@northwestern.edu (C.K.B.); 3 Anita Zucker Center for Excellence in Early Childhood Studies, University of Florida, Gainesville, FL 32611, USA

**Keywords:** executive function, early experience, language, birth weight, regression trees, network analysis

## Abstract

**Highlights:**

**Public health relevance—How does this work relate to a public health issue?**

- Executive function (EF) skills in childhood and adolescence predict later academic performance and mental health, and this work demonstrates their associations with aspects of the prenatal environment, particularly birthweight.

**Public health significance—Why is this work of significance to public health?**

- This study employs a multimethod design to disaggregate associations with distinct early experiences to identify important predictors of children’s later EF skills.

**Public health implications—What are the key implications or messages for practitioners, policy makers and/or researchers in public health?**

- Public health initiatives targeting birth weight and its antecedents may have positive impacts on children’s cognitive development, with downstream effects on academic performance and mental health.

**Abstract:**

The healthy development of executive function (EF) skills in childhood and adolescence provides a crucial foundation for later outcomes, from mental and physical health to academic achievement and socio-emotional functioning. Although meta-analyses have identified associations between children’s EF skills and early experiential factors, these are often examined in terms of broad categories (e.g., socioeconomic status) or cumulative risk factors (e.g., adverse childhood experiences; ACEs). The current study leverages longitudinal data (*N* = 1295) from six cohorts from the Environmental influences on Child Health Outcomes (ECHO) program to identify unique associations among specific prenatal, perinatal, and early life conditions and three specific EF skills (inhibitory control, working memory, and cognitive flexibility) measured later in childhood. Indicators of socioeconomic status, birth outcomes, parental characteristics, and pre- and post-natal exposures to alcohol and tobacco were measured before age 6 years. EF skills and language were measured from ages 6–15 years (*M* = 9.95, *SD* = 1.92), using measures from the NIH Toolbox. Two complementary statistical methods, a psychological network approach and regression trees, were employed to disaggregate early predictors of EF development. Results from both methods converged to suggest that specific early conditions showed associations with specific EF skills, and that higher birth weight (independent of pre-term status) was a stronger predictor of better EF skills than other early conditions. Neither approach showed meaningful associations between EF skills and maternal ACEs. Birth weight appears to serve as a particularly sensitive summary index of prenatal influences on EF development.

## 1. Introduction

Executive function (EF) skills are a set of brain-based attention-regulation skills that are important for intentional, goal-directed problem solving, e.g., [1]. These skills include inhibitory control (suppressing attention to distractors), working memory (holding information in mind and manipulating it), and cognitive flexibility (flexibly shifting attention), as well as the *hot EF* skills of regulating emotions, resisting temptations, and flexibly adjusting one’s motivational tendencies, e.g., [2,3,4]. Together, EF skills make it possible to pay attention, keep goals and plans in mind despite distractions and interference, and consider alternative solutions. As such, they provide an essential neurocognitive foundation for intentional learning, deliberate reasoning, and more generally, flexible adaptation to the challenges of daily living.

Individual differences in EF skills measured in childhood have been found to predict a range of later developmental outcomes, with better EF skills associated with better academic achievement and social–emotional functioning, e.g., [5,6], as well as better physical health and higher socioeconomic status (SES) [7]. Systematic meta-analyses suggest these predictions are robust and observed in a wide range of countries [6,8,9,10,11,12]. By contrast, difficulties with EF skills are prominent in a wide range of neuropsychiatric and neurodevelopmental conditions, including attention-deficit/hyperactivity disorder (ADHD) and autism spectrum disorder [13]. The ubiquity of EF difficulties across conditions suggests that the disruption of EF development may be a common consequence of many kinds of developmental perturbation (e.g., genetic, environmental, or epigenetic disturbances; or cognitive, emotional, or social deprivation). For this reason, the presence of EF difficulties can be considered a transdiagnostic indicator of atypical development in general, e.g., [14].

Children’s use of language (i.e., inner speech) is believed to facilitate EF skills, (e.g., [1,15,16]), and there are strong positive concurrent, (e.g., [17]) and longitudinal relations, (e.g., [18]) between language and EF skills across development. Indeed, disrupting inner speech via articulatory suppression impairs children’s and adults’ performance on EF tasks [19,20], suggesting that language plays an instrumental role in accomplishing top-down self-regulation. But whereas basic cognitive functions such as language and memory emerge relatively early in development, there are major increases in the efficiency and effectiveness of EF skills that continue into early adulthood, e.g., [21,22]. This more protracted development of EF skills corresponds to the similarly protracted development of higher-order brain networks involving the prefrontal cortex that are built upon and regulate earlier-developing networks, e.g., [23,24,25]. Due to their protracted development across the lifespan and their hierarchical relation with the lower-order skills that they regulate in a top-down fashion, the development of EF skills might be particularly susceptible to influences from early experiences.

Understanding early predictors of the healthy development of EF skills, and how they interact, informs policy and practices designed to support this development. Key early-experience predictors of EF development include socio-economic status SES [26], preterm birth and birth weight [27], and maternal mental health [28], among others. Additionally, even more distal predictors such as maternal adverse childhood experiences (ACEs) have been found to be associated with children’s EF skills [29], with evidence suggesting mediation through stress and parenting practices, including maltreatment [30].

To date, most existing studies of early experience and EF skills utilize cross-sectional designs and model a limited number of early life exposures or use aggregate measures of these exposures, such as SES or ACEs. For example, SES is often defined in psychological research as a singular category, indexed by parental education and family income, but this category likely reflects numerous distinct features of children’s environments across multiple levels of analysis, e.g., [31,32]. These features include, but are not limited to, individual-level constructs like exposure to stress or toxins, household-level constructs like parental income and education, and neighborhood-level constructs like poverty and safety, e.g., [33]. As another example, ACEs are often modeled as cumulative risk [34], but there may be variability in how specific ACEs differentially impact development, e.g., [32,35]. Finally, features of children’s environments likely interact longitudinally as development unfolds. For example, birth weight is predicted by preconception and prenatal experiential factors such as air pollution exposure [36], maternal mental health during pregnancy [37], maternal education [38], and neighborhood disadvantage [39]; birth weight, in turn, positively predicts numerous developmental outcomes, including EF skills, e.g., [27].

A first step in understanding how pre- and postnatal experiences influence EF development is to identify unique associations among specific prenatal, perinatal, and early life adversities and specific later EF skills, and to examine the relative strength of these associations. The current study leverages longitudinal data (*N* = 1295) from six cohorts from the National Institutes of Health (NIH) Environmental influences on Child Health Outcomes (ECHO) program [40]. ECHO is a longitudinal observational pediatric cohort consortium of over 50,000 children and their caregivers at study sites across the US and Puerto Rico. ECHO’s primary aim is to investigate early physical/chemical, lifestyle, and psychosocial exposures and children’s physical, mental, and social health outcomes. For the current study, the following early life experiences were measured before age 6 years: indicators of socioeconomic status, birth outcomes, family mental health (e.g., maternal depression, family psychiatric history), and pre- and post-natal toxin exposures (e.g., prenatal tobacco use and secondhand smoke exposure). Additionally, because language skills are known to relate to both children’s SES and their EF development [41,42], we also measured vocabulary as a predictor of EF skills, as a proxy for exposure to enriching and cognitively stimulating home environments. EF skills and vocabulary were measured from ages 6–15 years (*M* = 9.95, *SD* = 1.92), using age-adjusted scores from measures from the NIH Toolbox Cognition Battery [43,44,45]. We chose to measure EF in this age range in order to keep our measures of early life experiences temporally distinct from our measures of EF, and because we were primarily interested in detecting associations with EF skills that might reflect enduring influences across childhood and into adolescence. Specifically, we used the Flanker Inhibitory Control and Attention Test (Flanker) to measure inhibitory control of attention to distracting stimuli; the Dimensional Change Card Sort (DCCS) to assess cognitive flexibility; the List Sorting Working Memory (LSWM) Test to measure working memory; and the Toolbox Picture Vocabulary Test (TPVT) to assess vocabulary, which is a good proxy for broader language ability in this age range, e.g., [46]. To disaggregate and compare specific early predictors of EF development, we used two complementary statistical methods: a psychological network approach, e.g., [47]; and regression trees, random forests, and conditional inference trees, e.g., [48]. Network analyses can reveal correlations between predictors while controlling for all other variables, reducing the impact of masking and identifying the remaining connections. Meanwhile, regression trees allow deep exploration of contextual associations, uncovering whether each predictor has distinct relations with EF skills across different levels of another predictor. We hypothesized that specific early life experiences would have distinct associations with EF skill development that vary in magnitude, and we also expected, based on prior research, that EF skills would be most strongly related to language measured concurrently.

## 2. Materials and Methods

### 2.1. Participants

As part of the Environmental influences on Child Health Outcomes (ECHO) study, we pooled data from the six cohorts for whom we had the most complete data on our variables of interest; see Table 1 for demographic information about the combined sample. These six cohorts were selected by systematically assessing the fraction of missing information (FMI) across all thirty-four ECHO cohorts with any available EF data, aiming to balance generalizability with needing to impute more data. See Supplemental Materials for more details on cohort selection (Table S1, Figures S1 and S2). This resulted in 1295 participants who were included in analyses (50.7% female). Participants were described by parents as 43.7% White, 37.7% Black, 2.2% Asian, 0.2% Native Hawaiian/Pacific Islander, 2.2% American Indian/Alaskan Native, 9.9% Multiracial, and 1.9% had no reported race. Independently of those categories, 16.6% were identified as Hispanic by parents. Participants’ parental education level was 8.3% less than high school, 24% high school degree, 21.9% some college/trade school, 20.6% bachelor’s degree, 16.1% master’s or professional/doctorate degree, and 9.1% were missing education information. In terms of participants’ household composition, 67.5% were in a two-parent household and 31.4% were in a single-parent household, with 1.2% missing. Finally, 34.1% of participants’ family income level was less than $30,000 per year, 13.7% was between $30,000 and $49,000, 13.7% was between $50,000 and $74,999, 17.5% was greater than $75,000, and 21.1% were missing income information. Sociodemographic characteristics between participants in included and excluded cohorts were broadly similar, although excluded cohorts had a larger proportion of missing information, which led to higher percentages of participants reporting the presence of targeted early life experiential factors in included cohorts (see Table 1). The study protocol was approved by the single ECHO institutional review board, WCG IRB. Written informed consent or parent’s/guardian’s permission was obtained along with child assent as appropriate for the ECHO Cohort Data and Biospecimen Collection Protocol participation and for participation in specific study sites.

### 2.2. Measures

#### 2.2.1. Early Life Experiences

**Figure 1:**
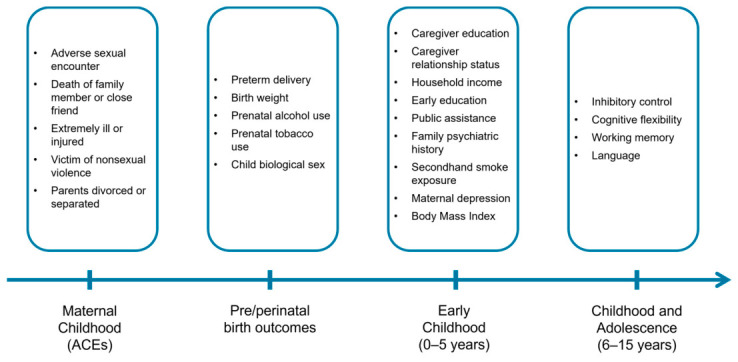
Timeline of early experiences across the lifespan. Note that data for all early experiences were collected during the prenatal period through age five years. This figure instead depicts the ages at which these exposures actually occurred.

Early life experiences were measured within and across the categories of SES indicators, birth outcomes, family mental health, and pre- and postnatal toxin exposures. Data were collected through self-report by primary caregivers, who were almost exclusively biological mothers, during the prenatal period through age 5 years. See Figure 1 for a timeline depiction of the ages at which early experiences occurred.

SES indicators included caregiver education level, caregiver relationship status, household income, early childcare education, and family reliance on public assistance. For education level, relationship status, and household income, data from the most recent time point (i.e., when their child was the oldest) were used if more than one report was available. However, for early childcare education and public assistance, the response was coded as “yes” if participants reported utilizing these services at any time point.

Birth outcomes included preterm delivery and birth weight, each measured by the most reliable available source (maternal medical record, followed by childbirth/neonatal medical record, followed by maternal self-report). Preterm delivery was coded as “yes” if the child’s birth occurred earlier than 37 weeks, and birth weight was measured in grams. Family mental health was assessed using measures of maternal depression (PROMIS-D *T*-score; [49]), family psychiatric history (whether any first-degree relatives had ever been diagnosed with a psychiatric disorder), and maternal Adverse Childhood Experiences (ACEs; adverse sexual encounter, death of family member/close friend, extremely ill/injured, victim of nonsexual violence, parents divorced/separated). For maternal depression, the highest score was chosen if there were multiple reports across infancy and early childhood. However, for family psychiatric history and maternal ACEs, the response was coded as “yes” if participants reported the presence of these risk factors at any time point.

Pre- and postnatal toxin exposures included were secondhand smoke exposure, maternal prenatal alcohol use, and maternal prenatal tobacco use [50]. If participants reported the presence of these toxin exposures at any time point, the response was coded as “yes.” Finally, childhood body mass index (BMI) was measured by dividing weight (*kg*) by height squared (*m^2^*), and the most recent time point (i.e., when the child was the oldest) was chosen if more than one report was available.

#### 2.2.2. Cognitive Measures

Children were administered the NIH Toolbox cognitive assessments via electronic tablet during middle childhood and adolescence (*M* = 9.95 years, *SD* = 1.92, range = 6.00–15.41). This included the TPVT to measure language (vocabulary), the Flanker task to measure inhibitory control, the DCCS to measure cognitive flexibility, and the LSWM Test to measure working memory. From these measures, we utilized the age-adjusted standard scores, norm-referenced such that a score of 100 represents the approximate mean of the general population of children at the examinee’s age, with a corresponding standard deviation of 15.

### 2.3. Analytic Plan

All analyses were conducted in R (version 4.5.0) [51], using packages listed below.

#### 2.3.1. Network Analysis

The network model included all 23 variables described in the *Measures* section. Because our data included both categorical and continuous variables, we estimated the network structure using the Mixed Graphical Model (MGM) with the R package *mgm* (version 1.2-15) [52]. In network models, variables are represented as nodes, and the connections between nodes are edges, reflecting direct conditional associations between two nodes. MGMs are specifically designed to accommodate variables of mixed types (e.g., continuous, categorical, and count) by estimating conditional associations using nodewise generalized linear models; in such models, associations between continuous variables are interpreted as partial correlations and associations involving categorical variables are interpreted as averaged regression coefficients [53]. To control for potential spurious associations, *mgm* applies a Least Absolute Shrinkage and Selection Operator (LASSO) regularization penalty. LASSO shrinks all edge weights toward zero and eliminates smaller edges by assigning them to exactly zero, promoting a more sparse network structure. The extent to which LASSO shrinks edge weights is determined by the parameter *lambda*, which can be selected using the Extended Bayesian Information Criterion (EBIC) or cross-validation. We selected EBIC for model estimation, setting its hypertuning parameter *gamma* to the recommended default value of 0.25 [52].

Before constructing the network model, we examined the potential for statistical redundancy among variables using the *goldbricker* function from the R package *networktools* (version 1.6.0) [54]. This procedure identifies pairs of variables that might be statistically redundant based on their correlation patterns with other variables in the network. Additionally, a number of variables included in the network model had missing data, which the *mgm* package (version 1.2-15) cannot handle, so we used multiple imputation using the R package *mice* (version 3.18.0) [55]. Following previous studies [56,57], we created 10 imputed datasets and retained only those edges that appeared in the estimated networks of at least nine of the ten imputed datasets. Lastly, we assessed the stability of edge estimates using bootstrapping routines implemented in the R package *bootnet* (version 1.6) [58]. The resulting network model was visualized via the R package *qgraph* (version 1.9.8) [59], with the layout determined by the Fruchterman–Reingold algorithm. This force-directed layout algorithm arranges nodes based on their connectivity, such that nodes with a greater number and higher strength of connections appear closer together and less connected nodes are positioned farther apart. Because the network includes both continuous and categorical variables, and the resulting edge weights are not on a common scale, we do not report quantitative values of edge weights. See Supplemental Methods for additional information about network analysis and missing data handling.

#### 2.3.2. Regression Trees

We used regression trees to examine the contextual patterning of EF skills in middle childhood and adolescence across levels of prenatal, perinatal, and early childhood predictors. Regression trees repeatedly split the data according to the level of a predictor that most differentiates the resulting subgroups on the outcome, repeating until a statistical stopping criterion is reached and no significant differences can be found. As a result, regression trees treat all predictors as potential moderators and allow for nonlinearities and interactions of complexity limited only by sample size within each subgroup. Here, we first applied random forests, which use an ensemble of regression trees built from random subsets of predictors and observations, to rank early life variables by their importance in predicting EF. We utilized the *ranger* package (version 0.17.0) [60], which does not allow multivariate outcomes; therefore, we treated each EF score as a separate outcome and calculated permutation importance to quantify each predictor’s predictive value within the random forest. See Supplemental Methods for additional information about regression trees and missing data handling.

Random forests allow highly accurate prediction and importance quantification, but their complexity makes them difficult to interpret. To produce interpretable tree-based models of our data, we utilized conditional inference trees *ctree*; ([61]), implemented within the *partykit* package (version 1.2-24) [62]. The *ctree* algorithm searches each predictor for the threshold (for continuous or ordered categorical predictors) or sets of categories (for unordered categorical predictors) that yield the lowest *p* value for outcome differences, and then selects from among those predictor-specific splits using the lowest *p* value criterion, terminating when no *p* value passes a Bonferroni correction. In short, every *ctree* split represents a Bonferroni-corrected statistically significant difference, and *ctree* stops partitioning when there are no more such differences.

## 3. Results

### 3.1. Descriptive Correlations

**Figure 2:**
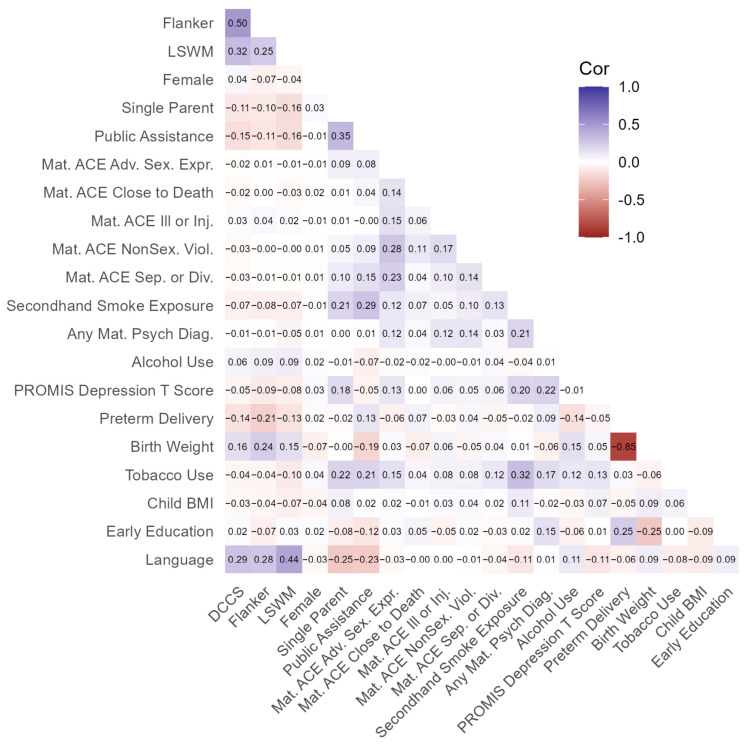
Correlation table with imputed data for all study variables except factors with greater than two levels. For pairs of continuous variables, values represent Pearson correlations; for continuous-binary pairs, values represent point-biserial correlations; for binary-binary pairs, values represent phi (φ) coefficients.

We first conducted a correlational analysis among all study variables except factors with greater than two levels (i.e., caregiver education and household income) to describe the overall pattern of associations among study variables (Figure 2). Results showed numerous intercorrelations among variables; however, our primary study aim was to examine the way in which multiple early experiences interact to predict later EF skills. Thus, our primary results involve (1) a network analysis, which controls for the influence of all other predictors to reduce masking, and (2) regression trees, which reveal how the associations between predictors and EF might vary across levels of other predictors.

### 3.2. Network Analysis

**Figure 3:**
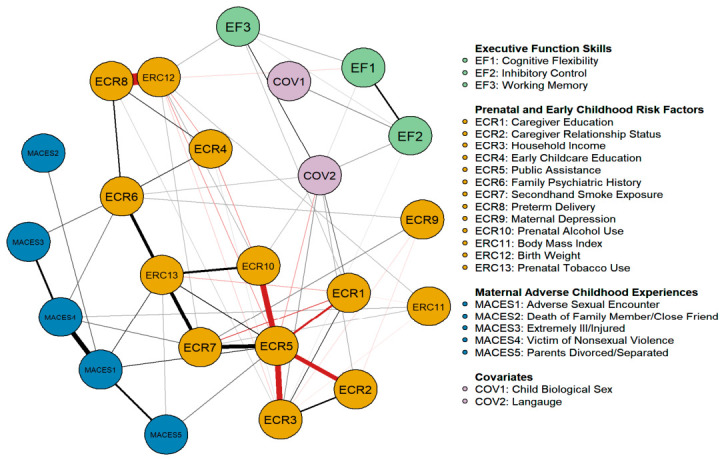
Psychological network model of executive function (EF) skills, prenatal and early childhood risk factors, maternal adverse childhood experiences (ACES), and covariates. *Note*. The circles in this figure (i.e., nodes) represent the variables that were measured and included in the network analysis. The black and red lines connecting the nodes are edges that represent positive and negative regularized conditional associations, respectively. The strength of the association between nodes is indicated by the thickness of the line, with thicker lines reflecting stronger estimated associations. However, because the network includes different types of variables (i.e., continuous and categorical), the edge weights are not on a common scale and therefore should not be interpreted as directly comparable across all edges. Edge thickness should be interpreted only in relative terms within the same type of variable pairing.

Figure 3 shows the resulting network structure of EF skills, prenatal and early childhood risk factors, maternal ACEs, and covariates. No redundant variables were identified, based on a minimum zero-order correlation threshold of 0.5 and a maximum proportion of significantly different correlations set at 0.25. While all three EF skills were correlated with one another and with language as measured by the TPVT, they exhibited distinct associations with early childhood risk factors. Cognitive flexibility (DCCS) was negatively associated with preterm delivery, indicating that youth born preterm tended to perform more poorly on the DCCS task in childhood and adolescence. Inhibitory control (Flanker) was positively associated with household income, and working memory (LSWM) was positively associated with both parental education and birth weight. Additionally, boys performed better on the Flanker compared to girls. There were no significant direct associations between any EF skills and maternal ACEs.

As expected, language (vocabulary) emerged as a highly connected node within the network. In addition to being associated with all three concurrently measured EF skills, language was positively associated with multiple SES variables, including higher caregiver education, higher household income, and a two-parent household status, and negatively associated with receiving public assistance. Language was also positively associated with both prenatal alcohol exposure and having a family member diagnosed with a mental health condition.

Although preterm delivery and birth weight were strongly correlated, they exhibited unique associations with other variables in the network. Birth weight was positively associated with the LSWM, whereas preterm delivery was negatively associated with the DCCS. Moreover, birth weight was negatively associated with household income and receiving public assistance, whereas preterm delivery was positively associated with family psychiatric history. Both variables were associated with early childcare education, such that children born prematurely or with lower birth weight were more likely to have attended an early childhood learning program.

Among the SES variables, parental education was negatively associated with secondhand smoke exposure and prenatal tobacco use, indicating that more educated parents were less likely to report these risks. Higher household income was associated with a greater likelihood of attending an early childcare learning program and with maternal reports of parental separation/divorce during their own childhood, as well as with lower birth weight. Parents with higher education, higher income, and who were partnered or married were less likely to report receiving public assistance. Lastly, maternal depressive symptoms were higher in households with lower income.

In a follow-up sensitivity analysis, we re-estimated the network including an ECHO cohort identifier as a node and assessed its relations with other modeled variables and how associations among those variables changed with this addition. Cohort was highly connected to almost every variable in the network, including DCCS, Flanker, and LSWM. With cohort included, connections between EF and other variables were identical to when cohort was not included, except that a new association emerged between working memory and caregiver relationship status when cohort was included, such that youth from two-parent households performed better on LSWM.

### 3.3. Regression Trees

**Figure 4:**
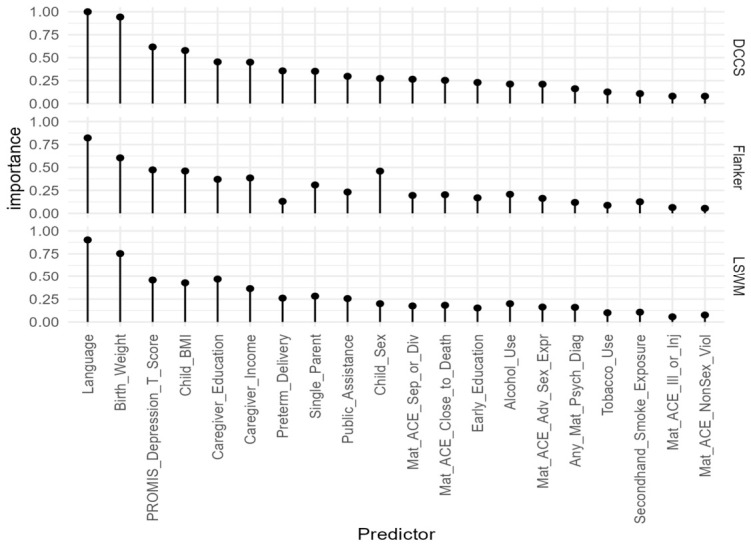
Random forest variable importance predicting each executive function (EF) score. *Note*. ACES = Adverse Childhood Experiences; BMI = Body Mass Index; DCCS = Dimensional Change Card Sort; LSWM = List Sort Working Memory; Mat. = Maternal.

Random forest analyses predicting DCCS (cognitive flexibility), Flanker (inhibitory control), and LSWM (working memory) consistently identified the TPVT (language) as the most important predictor, followed by birth weight (see Figure 4). Further rank-ordering of predictor importance varied by outcome; for DCCS and Flanker, the next most important predictors were maternal depression and child BMI, while for LSWM the next most important predictors were caregiver education, followed by maternal depression and child BMI. Child sex was more important for predicting Flanker than for other outcomes, and preterm delivery was less important for Flanker than other outcomes.

**Figure 5:**
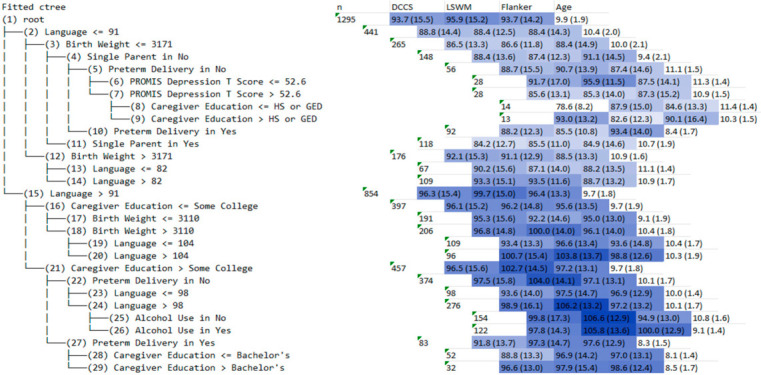
Conditional inference tree predicting three EF scores (Dimensional Change Card Sort [DCCS]; Flanker; and List Sort Working Memory [LSWM]) as a multivariate outcome. <, <=, >=, and > are used to indicate thresholding for splits of continuous and ordered categorical predictors. Each outcome is separately color-coded across all nodes, with darker colors indicating higher mean scores for that predictor.

In the multivariate *ctree* with DCCS, Flanker, and LSWM as outcomes (Figure 5), the first split was on language, with children with TPVT standard scores greater than 91 scoring higher on all EF measures (mean standard scores: DCCS = 96.3, LSWM = 102.7, Flanker = 97.2) than those with scores less than or equal to 91 (mean standard scores: DCCS = 88.8, LSWM = 88.4, Flanker = 88.4). This first split represents the most statistically significantly different single split identified by *ctree* within the full sample, dividing the sample into lower-language and higher-language subsamples. Within each subsample, further splits appeared based on language (higher language associated with higher EF scores), birth weight (higher birth weight associated with higher EF scores, with thresholds of 3171 and 3110 g), preterm birth (associated with lower scores), and caregiver education, with varying split levels for education and language across the tree. Generally, higher caregiver education was associated with higher EF scores, although within one smaller subsample (node 7; *n* = 27), higher education was associated with higher DCCS and Flanker scores but *lower* LSWM scores. This subsample was characterized by low language and birth weight, no preterm delivery, and married caregivers with above-average depression scores.

Prenatal alcohol use was used for splitting only in the higher-language subsample of the multivariate *ctree* resulting from the first split, specifically within the subset of this higher-language subsample further defined by higher education (Bachelor’s degree or higher), no preterm delivery (*n* = 374), and higher language scores (>98; *n* = 276). Therein, prenatal alcohol use was associated with lower DCCS and LSWM scores but higher Flanker scores. Note that subsample numbers, as well as others in Figure 5, sometimes do not exactly sum to the total for the parent node, because some predictors were imputed and the same child may be classified into different terminal nodes if imputed predictors were used for splitting.

In a follow-up sensitivity analysis, we re-estimated the *ctree*, including an ECHO cohort identifier as a predictor and compared the predictive strength (*R^2^*) of the *ctree* before and after this addition for each outcome. This addition yielded only slightly higher predictive power (*R^2^* = 0.323, 0.346, and 0.256 for DCCS, LSWM, and Flanker, respectively) than when cohort was not included (*R^2^* = 0.322, 0.336, and 0.227, respectively).

## 4. Discussion

Results from our two complementary analytical approaches underscore the importance of examining heterogeneity within children’s early life experiences. Instead of simply modeling early experience as one construct (e.g., cumulative risk or SES), we were able to examine the relative importance of specific early life experiences, accounting for other predictors in a network analysis and allowing for complex, nonlinear interactions with regression trees. Moreover, we examined all three commonly studied aspects of cool EF (cognitive flexibility, working memory, and inhibitory control). Across our analytical approaches, results converged to suggest that early experiences have complex, multidimensional predictive relations with specific EF skills, with birth weight emerging as an especially important predictor. As expected, there was also strong association between EF skills (especially working memory) and language measured concurrently. In the conditional inference tree, language and birth weight were used for splitting at multiple levels of depth, suggesting the general importance of these variables across contexts. Neither approach revealed meaningful associations between EF and specific maternal ACEs measured individually. Although, in theory, operationalizing early experiences as separate variables evidently offers increased insight into the complex interplay between birth outcomes, socio-economic indicators, and family mental health, it is possible that maternal ACEs were not important predictors of children’s EF skills in our study because they are indeed best measured as cumulative risk within this context.

Results regarding children’s language (TPVT score) converged across analytic methods: This variable ranked in the random forest analysis as the most important predictor, was revealed to be the first split in the multivariate *ctree*, and was the only covariate that was positively associated with all three cool EF skills in the network analysis. Importantly, thresholds used for splitting were below or near the average for the NIH Toolbox normed average (*M* = 100, *SD* = 15), suggesting that the relation between language and EF is most prominent at these levels. Language skills may be necessary for the successful implementation of EF skills through the use of potentially silent self-directed speech, i.e., “inner speech”; e.g., [1,15,16]. As children learn to internalize rules using language, they can more effectively guide their own attention toward long-term goals. Language was also related to multiple early life experiences in the network analysis, several of which were within the domain of SES (e.g., caregiver education, household income, and public assistance). Children from higher SES families are known to experience greater cognitive stimulation in the home environment, which improves language skills, in turn resulting in better EF skills [42]. It is important to note, however, that language skills were measured concurrently with EF skills in our sample, and might reflect bidirectional influences, and future studies should investigate longitudinal relations between these two cognitive processes.

After language, our second-most important predictor for children’s EF skills, according to random forest analysis, was birth weight. In the multivariate *ctree*, birth weight was revealed as the most statistically significant split for those with low language, and the most statistically significant split for those with high language and less than a Bachelor’s degree. These splits appeared at weights near 3150 g, which is higher than the World Health Organization criterion of <2500 g for low birth weight classification, indicating that any birth weight below average may confer developmental risk. Finally, results from the network analysis converged with those from the *ctree* and random forest analysis, and these results further situated birth weight in the context of other early life conditions and in relation to specific EF skills. Birth weight was related to working memory as well as numerous early childhood risk factors: household income, early childcare education, public assistance, secondhand smoke exposure, preterm delivery, and prenatal alcohol use. It is notable that birth weight was revealed to be a more important predictor of EF skills than other indicators of prenatal development, namely preterm delivery, alcohol exposure, and tobacco exposure. Importantly, birth weight presumably reflects the culmination of a series of complex interactions across prenatal development, so risk factors such as preterm delivery or alcohol exposure may only predict later cognitive development through mechanisms that more prominently manifest in low birth weight.

Early environmental indicators were generally related to EF in expected ways in the *ctree*, with higher caregiver education, for example, associated with higher EF scores. Although prior work has tended to consider SES as a single, aggregate construct, our study reveals heterogeneity among separate SES indicators. For example, we found that caregiver education was the most statistically significant split for children with high language scores, whereas it was only relevant for a very small subset of children (*n* = 27) with low language scores–those with low birth weight, from a two-parent household, and with high maternal depression. One surprising finding was that within a narrowly defined subsample of higher-educated mothers of children not born preterm and with average language scores or higher (*n* = 276), prenatal alcohol use was associated with higher Flanker (inhibitory control) scores. It should be noted, however, that the self-report question used to obtain our data regarding prenatal alcohol use did not distinguish between heavy, light, or even extremely infrequent alcohol use, and we recommend this finding be interpreted with considerable caution. Recent work has shown that even low levels of prenatal alcohol use can have detrimental impacts on children’s neurodevelopment [63].

The *ctree* results also corroborated and added to our understanding of complex associations among concurrent working memory and language, and earlier maternal depression. Maternal depression emerged as a significant predictor in our study; in the subset of our sample with low language and birth weight, no preterm delivery, and married caregivers, children of caregivers reporting above-average depression scores demonstrated lower performance on the LSWM measure of working memory (about ⅔ of a standard deviation with respect to the test’s norms) and the DCCS measure of cognitive flexibility (about ⅓ of a standard deviation). These associations align with previous research linking maternal depression to lower levels of children’s cognitive flexibility, e.g., [64,65]. Furthermore, our study demonstrated a contextual association between maternal depression and children’s language. This underscores the potential effect of parents’ cognitive stimulation and engagement with their children on the development of language and EF skills, including both working memory and cognitive flexibility, e.g., [66,67]. Prior work suggests poor maternal mental health is associated with less cognitively stimulating caregiving environments and more negative parenting behaviors, both of which offer fewer opportunities for young children to practice and develop foundational language and EF skills. These findings reinforce the suggestion that interventions targeting maternal depression could yield dual benefits—improving caregivers’ mental health while also potentially enhancing their children’s EF skills.

The complexity of our *ctree* reflects the “depth” of interactions identified in our data. Our ability to detect such interactions was limited by the statistical power available in our data, and focused on the predictors that yielded the greatest differences between terminal nodes. Notably, several potential predictors included in *ctree* were not found to be significant; for example, maternal ACEs, public assistance, secondhand smoke, and BMI. These predictors were potentially (a) masked by other variables with more significant differences when building the *ctree*; (b) not important for predicting childhood EF skills; or (c) both. In our data set, maternal ACEs were the most temporally distant variables from measured childhood EF outcomes, which may be why maternal ACEs were not found to be significant predictors of children’s EF skills. In contrast, language skills (measured contemporaneously with EF skills) were the first split in the *ctree*. The relative unimportance of ECHO cohort in the *ctree* was likely due to differences between cohorts in our other predictors, which resulted in splits on cohort rather than other, collinear predictors when cohort was included (see Supplemental Table S2). We excluded the cohort identifier from our presented *ctree* in the interest of presenting a model whose variables and relations can be interpreted outside the context of the ECHO study. As described in the Results, little predictive power was lost in doing so.

Network analysis, our other statistical approach, avoids the issue of masking present in *ctrees*, allowing us to identify correlations between various early life predictors while controlling for confounders. Network results largely converged with results from the *ctree* and random forest analyses, but also revealed several unique associations between specific early life experiences and specific EF skills, which contributes to a more nuanced characterization of the potential influence of early experiences. Inhibitory control was positively associated with household income; working memory was positively associated with both caregiver education and birth weight; and cognitive flexibility was negatively associated with preterm delivery. Meanwhile, language was positively associated with all three EF skills.

Results from the network analysis also revealed how specific early experiences were related to one another, and to language, instead of only modeling associations between early experiences and later EF skills, as in the *ctree* and random forest analyses. For example, language was positively associated with multiple early life experiences, including caregiver education, caregiver relationship status, household income, and public assistance. Additionally, birth weight and preterm delivery were both positively associated with early childcare education, but only birth weight was positively associated with household income and negatively associated with public assistance, and only preterm delivery was positively associated with family psychiatric history. These results, in addition to the previously discussed finding that birth weight and preterm delivery were related to distinct EF skills in the network, highlight the necessity of disaggregating early experiences, even those that may appear similar at first glance.

In network analyses, parental education was negatively related to secondhand smoke exposure and to prenatal tobacco use, but not the other measured toxin exposure, prenatal alcohol use. Lower household income was associated with more maternal depressive symptoms, and higher household income was associated with early childcare education. Although some children may attend early childcare education programs like Head Start specifically due to low socioeconomic status, this finding indicates that, in our sample, early childcare education programs were likely to be private programs only available to those with higher household incomes. Finally, the network analyses revealed a few surprising correlations; higher income was related to lower birth weight, and higher language scores were related to prenatal alcohol use and family psychiatric history. These unexpected findings might reflect issues in measurement (i.e., which participants were more likely to accurately report alcohol use or psychiatric history), or they might reflect genuine relations. As these results contradict previous studies, further research is necessary to clarify how these distinct early experiences might interact to predict later EF skills. Similar to our *ctrees*, we also conducted a sensitivity analysis which included an ECHO cohort identifier in the network model. Cohort was revealed to be a highly connected variable, showing associations with nearly all other variables in the model (see Supplemental Table S2). However, its inclusion did not substantially change the key associations among EF skills and early childhood risk factors, so we again decided to present a simplified model that is more generalizable outside the context of the ECHO study.

Our study has numerous strengths; most notably the use of a large (*N* = 1295) dataset drawn from six different cohort study sites from across the United States, increasing the generalizability of our findings. In order to draw precise conclusions about the relative influence of multiple indicators of socioeconomic status, birth outcomes, and parental mental health, it is necessary for the sample to have variability across all these domains, something that is often not possible in a small-scale convenience sample. Additionally, our complementary statistical approaches avoid limitations inherent in commonly used methods, allowing for more nuanced insights into the complex interactions among early life predictors. Specifically, network analyses reduce the impact of masking when controlling for variables, helping to identify the most important overall predictors while accounting for the others. And, relative to generalized linear models, regression trees offer a more appropriate method of handling the nested, cohort structure of our data [68]. Importantly, regression trees offer insight into heterogeneous predictor spaces, revealing how each predictor might have unique associations with specific EF skills across different levels of another predictor.

Despite these strengths, however, there are several limitations to note. First, language and EF skills were measured concurrently, and it is impossible to disentangle the likely direction of causality for this association. The link between language and EF skills might reflect shared domain-general cognitive processes, and/or it might reflect bidirectional influences between distinct cognitive processes. Second, even for the aspects of our study that do leverage longitudinal data (early life characteristics measured prior to EF outcomes), all analyses were associational, limiting causal inferences. For example, although birth weight showed the strongest influence among early life predictors on later EF skills, birth weight was itself related to many other experiential factors such as alcohol exposure and parental income. Whether birth weight’s association with later EF skills is caused by birth weight per se, or whether it is caused by a third variable influencing both birth weight and EF skill development, remains unknown. Although our set of variables covered many distinct aspects of early experience, there are numerous unmeasured variables (e.g., cognitive stimulation in the home or parenting quality) that might have influenced our results.

Third, we selected participants that had the most available data on our measures of interest to maximize data completeness. While this came at the cost of leveraging the full ECHO Cohort sample, we still maintained a large sample size compared to prior investigations of individual early life influences on childhood EF. We were similarly limited in our choice of measures for cognitive skills. For example, although we examined specific aspects of cool EF, we were not able to include any measures of hot EF skills, which could show different associations with early experiences, e.g., [13]. Moreover, we relied on a single test of vocabulary to represent language skills, and only one measure for each of the three canonical cool EF components of working memory, cognitive flexibility, and inhibitory control. These measures are not exhaustive, and results therefore may be specific to these tasks. Differences in relations with cool EF components should therefore be interpreted with caution; for example, that working memory was associated with parental education while inhibitory control was associated with household income. Future studies should further investigate the relation between birth weight and EF skills, given the predictive importance of birth weight in our sample. Many studies of birth weight and later cognitive development only examine birth weight within the context of preterm birth, e.g., [27], but our study demonstrates a potential dissociable effect of these two risk factors. Because birth weight is itself related to numerous other prenatal risk factors, interventions targeting these indicators to improve birth weight could positively impact children’s EF skills years later. Therefore, it is imperative that we uncover the mechanisms by which this relation unfolds across development.

The finding that maternal depression was a significant predictor of EF skills in a subset of our sample also warrants further study. Because maternal depression was treated as static, and simplified to only one score per child in our analyses, it is necessary for future work to explore longitudinal interactions and relations among depression and children’s EF skills, e.g., [64]. For example, examining the longitudinal relations among maternal depression, parenting, and children’s EF skills would offer insight into a potential pathway through which maternal depression might influence the development of children’s EF skills (e.g., [66]). This limitation holds true for the remaining early experiences as well, particularly for those that assess children’s exposure to harmful substances or behaviors. For example, children with reported secondhand smoke exposure at any time point were all coded as “yes”, so we could not evaluate differences in the frequency or severity of these exposures. However, by simplifying all variables into single scores and treating them similarly, we could incorporate a larger set of variables into our models and examine the full breadth of early experience.

## 5. Conclusions

The consequences of supporting the development of EF skills are potentially far-reaching, and may lead to improvements in children’s problem solving, emotion regulation, and school success, as well as increasing in their perspective taking, empathy, and prosocial behavior. Overall, the results of this study contribute to a more fine-grained characterization of the complex constellations of early exposures predicting children’s later EF skills. Instead of aggregating disparate early experiences into a broad summary statistic or assuming that one single variable can adequately represent the rich complexity of early experience, results from our multi-method study converged to suggest early life experiences exert complex, multidimensional influences on three critical cool EF skills, highlighting the need for future investigations into individual and combined pre- and postnatal exposures. Several unique associations emerged between specific early life conditions and specific EF skills. Whereas inhibitory control was positively associated with household income, and working memory was positively associated with both caregiver education and birth weight, cognitive flexibility was negatively associated with preterm delivery. Results from the *ctree* and random forest analyses, in particular, captured the way that early life conditions interact to predict EF skills, and might help identify subgroups of children who would benefit from a multi-pronged intervention approach that targets a combination of exposures.

## Figures and Tables

**Table 1:** Sample demographic information compared for included and excluded cohorts.

Characteristics	Included Cohorts (*N* = 1295)	Excluded Cohorts (*N* = 3982)
Child Sex		
Female	657 (50.7%)	1876 (47.1%)
Male	638 (49.3%)	2106 (52.9%)
Child Race		
American Indian or Alaska Native	29 (2.2%)	12 (0.3%)
Asian	28 (2.2%)	83 (2.1%)
Black	488 (37.7%)	570 (14.3%)
Native Hawaiian or other Pacific Islander	3 (0.2%)	7 (0.2%)
Multiple Race	128 (9.9%)	377 (9.5%)
Other Race	29 (2.2%)	139 (3.5%)
White	566 (43.7%)	2742 (68.9%)
Unknown	24 (1.9%)	52 (1.3%)
Child Ethnicity		
Hispanic	215 (16.6%)	645 (16.2%)
Non-Hispanic	1076 (83.1%)	3332 (83.7%)
Unknown	4 (0.3%)	5 (0.1%)
Caregiver Education		
Less than High School	108 (8.3%)	353 (8.9%)
High School Degree	311 (24%)	540 (13.6%)
Some College	283 (21.9%)	847 (21.3%)
Bachelor’s Degree	267 (20.6%)	845 (21.2%)
Master’s Degree or higher	208 (16.1%)	593 (14.9%)
Unknown	118 (9.1%)	804 (20.2%)
Household Income		
<$30,000	441 (34.1%)	465 (11.7%)
$30,000–$49,999	178 (13.8%)	349 (8.8%)
$50,000–$74,999	177 (13.7%)	375 (9.4%)
$75,000 or more	226 (17.5%)	933 (23.4%)
Unknown	273 (21.1%)	1860 (46.7%)
Caregiver Relationship Status		
Married or Cohabitating	873 (67.4%)	2382 (59.8%)
Not Married or Cohabitating	407 (31.4%)	593 (14.9%)
Unknown	15 (1.2%)	1007 (25.3%)
Early Childcare Education		
Yes	503 (38.8%)	416 (10.5%)
No	558 (43.1%)	252 (6.3%)
Unknown	234 (18.1%)	3314 (83.2%)
Public Assistance		
Yes	742 (57.3%)	805 (20.2%)
No	231 (17.8%)	482 (12.1%)
Unknown	322 (24.9%)	2695 (67.7%)
Family Psychiatric History		
Yes	309 (23.9%)	614 (15.4%)
No	762 (58.8%)	1117 (28.1%)
Unknown	224 (17.3%)	2251 (56.5%)
Secondhand Smoke Exposure		
Yes	253 (19.5%)	284 (7.1%)
No	530 (40.9%)	840 (21.1%)
Unknown	512 (39.5%)	2858 (71.8%)
Preterm Delivery		
Yes	314 (24.2%)	1124 (28.2%)
No	981 (75.8%)	2699 (67.8%)
Unknown	0 (0%)	159 (4.0%)
Prenatal Alcohol Use		
Yes	303 (23.4%)	329 (8.3%)
No	983 (75.9%)	1946 (48.9%)
Unknown	9 (0.7%)	1707 (42.9%)
Prenatal Tobacco Use		
Yes	170 (13.1%)	311 (7.8%)
No	1115 (86.1%)	2788 (70.0%)
Unknown	10 (0.8%)	883 (22.2%)

Note: Not all percentages add exactly to 100%, due to rounding error.

## Data Availability

Select de-identified data from the ECHO Program are available through NICHD’s Data and Specimen Hub (DASH). Information on study data not available on DASH, such as some Indigenous datasets, can be found on the ECHO study DASH webpage.

## Supplementary Materials

The following supporting information can be downloaded at: https://www.mdpi.com/article/10.3390/ijerph23070904/s1, Figure S1: Fractions of missing information (FMI) as cohorts are added for potential inclusion in analysis; Figure S2: Fractions of missing information for variances and covariances of predictors and outcomes in the analysis sample; Table S1: Complete List of ECHO Cohorts Included in Sample; Table S2: Sample demographic information and cognitive outcomes compared across the six included cohorts [60,69,70,71,72,73].
