# Supplementary material for: Specific Early Childhood Experiences Predict Executive Function Skills During Later Childhood and Adolescence: Evidence from the ECHO Cohort
Source: Int J Environ Res Public Health. 2026 Jul 15;23(7):904. doi: 10.3390/ijerph23070904 (PMC13409844; doi:10.3390/ijerph23070904)
Supplement: Supplementary file 1 [file ijerph-23-00904-s001.zip › ijerph-4315444-supplementary.pdf]

Supplemental Materials

Supplemental Methods

Subsample Selection. Thirty-four ECHO cohorts had data on all EF outcome measures (DCCS, Flanker, LSWM). Missingness rates on other analysis variables varied between cohorts, and given the exploratory nature of our analysis, we did not prioritize completeness on any specific set of other predictors. Instead, we sought to identify a set of cohorts with as little *overall* missing information as possible. To do this, we utilized a statistical quantity from missing data handling called *fraction of missing information* (FMI), which quantifies the change in estimation precision for a set of estimates. FMI is related to missingness rate, but not identical. If two variables are near-perfectly correlated (e.g., body weight in kilograms and pounds, rounded to the nearest integer), and one has complete data, the other can be missing a relatively high proportion of *data* and be missing relatively little *information*, because missing values can be predicted well from observed values. FMI can be calculated using multiple imputation or full-information maximum likelihood estimation.

We selected cohorts by assessing how FMI changed depending on which cohorts were included. FMI was used to select cohorts for analysis as follows:

1. The cohort with the lowest percentage of missing values on variables of interest was identified; this defined the initial cohort set for consideration.

2. Multiple imputation in *mice* was used to create 100 imputed data sets for this set of cohorts.
3. We used the *fmi()* function in the *semTools* package (Jorgensen et al., 2025) in R to calculate FMI based on a saturated model (i.e., estimating all means and covariances) [1]. The mean FMI was calculated separately for means, variances, and unique covariances, and recorded.
4. The cohort with the next lowest percentage of missing values was added to the cohort set for consideration.
5. Steps 2-4 were repeated until the final cohort set included all 34 cohorts.

For each cohort set considered (lowest % missing values, then the two lowest % missing values, etc.), this process yielded mean estimates of FMI for means, variances, and covariances. Absent general guidance on acceptable FMI, we visually inspected a plot of these statistics across cohort sets (Supplemental Figure S1). Here, we focused on FMI for covariances, which are crucial for network analysis, as *mice* does not account for potential interactions for the recursive partitioning methods and thus it would not be fitting to optimize for that method. Unsurprisingly, using only one cohort yielded the lowest FMI, but analyzing only a single cohort's data would not leverage the variety of ECHO cohorts, harming our generalizability. The next "jumps" in FMI are from 1 to 2 cohorts, then 3 to 4, then 6 to 7; optimizing generalizability over the potential risk of imputing slightly more data, we selected the six cohorts with the lowest proportion of missing values for inclusion in analysis. Supplemental

Figure S2 shows FMI for variances and covariances in the final analysis sample consisting of these six cohorts.

Random Forests and Missing Data Handling. Random forests consist of an ensemble of regression trees, built from random subsets of predictors and observations. The individual regression trees in random forests have a lax stopping criterion and split based on minimum prediction error in the resulting nodes, such that each tree "overtrains" to its data, then predictions are averaged across the tree. Variable importance is calculated by permutation: a predictor is randomly scrambled and prediction error is compared to that arising from intact data, quantifying importance as the resulting decrease in prediction accuracy. Here, we used the *ranger* package (Wright & Ziegler, 2017) with 500 trees [2], and calculated permutation importance as described by Breiman (2001) [3]. Because importance values are estimates, rather than statistical tests or quantifications of uncertainty, importance can be calculated after stacking the imputed data sets into a single long data set *à la* Rodgers et al. (2021) and running the random forest algorithm on the stacked data set [4]. Because regression trees require deeper analyses of subgroups, we increased the number of multiple imputations from 10 to 100 for regression tree analysis.

When applying *ctree* to the stacked data set, the statistical criterion will be biased towards excessive splitting because the sample size will appear larger as a function of the number of imputations (here, 100 times as large), and *ctree* has no built-in method for accounting for multiple imputation. To account for this, after applying the *ctree* algorithm to the stacked data set, we pruned nodes that were not significant after dividing the chi-squared test statistics by the number of imputations and re-calculating Bonferroni-corrected p values. Although this approach does not fully account for between- vs within-imputation variance

(Enders, 2010; Campion, 1989) [5,6], it yields asymptotically identical results as the number of imputations grows, indicating that it is not biased by stacking imputed data sets, and thus suffices here as an *ad hoc* multiple imputation correction.

Supplemental Table S1 *Complete List of ECHO Cohorts Included in Sample*

Cohort Name	*N*
Conditions Affecting Neurocognitive Development and Learning in Early Childhood (CANDLE)	471
Neonatal Neurobehavior and Outcome in Very Premature Infants (ECHO-NOVI)	222
Safe Passage Study (PASS)	210
Vitamin D Antenatal Asthma Reduction Trial (VDAART)	183
Programming of Intergenerational Stress Mechanisms (PRISM)	164
Vitamin C to Decrease Effects of Smoking in Pregnancy on Infant Lung Function (VCSIP)	45

Supplemental Figure S1. Fractions of missing information (FMI) as cohorts are added for potential inclusion in analysis.

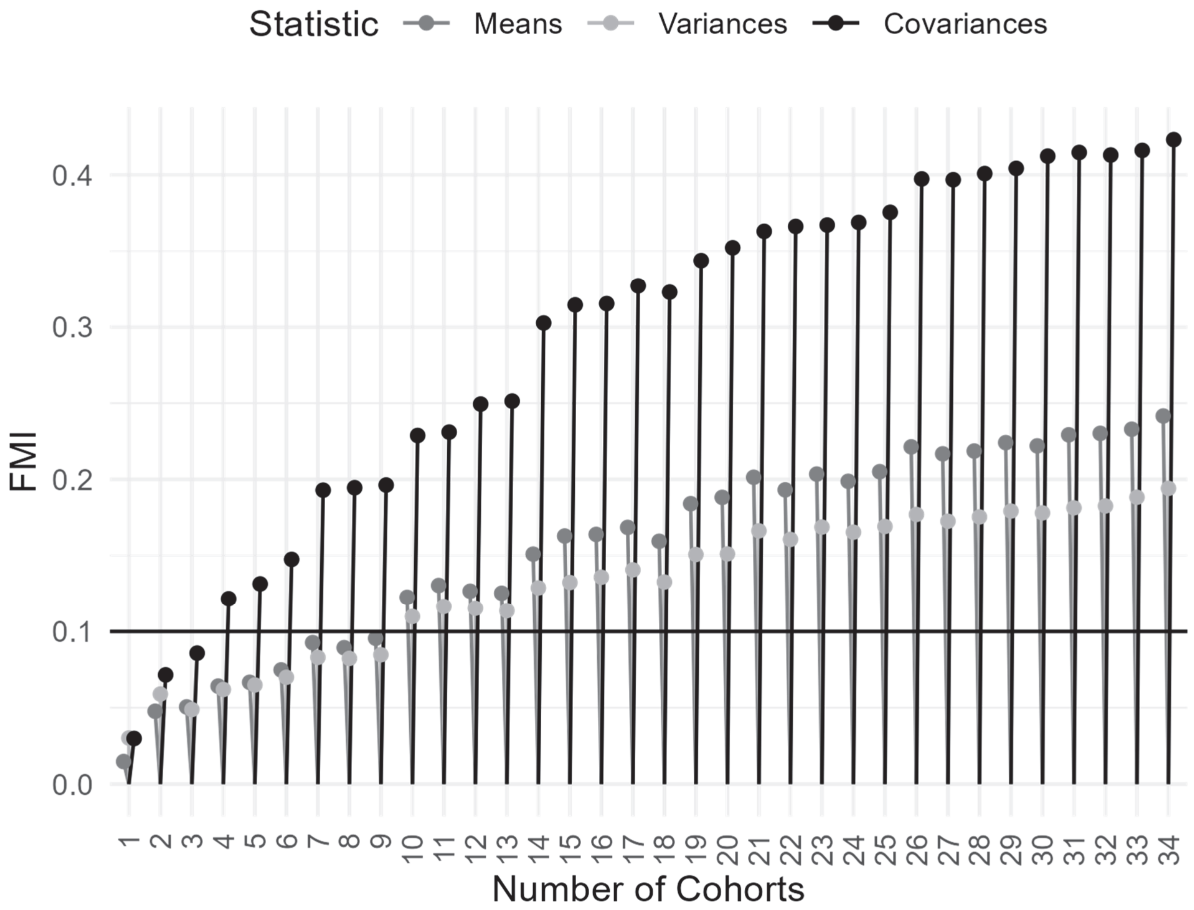

Note. Number 1 represents the cohort with the lowest overall percentage of missing values, 2 represents the two cohorts with the lowest percentage of missing values, etc.

Supplemental Figure S2. Fractions of missing information for variances and covariances of predictors and outcomes in the analysis sample.

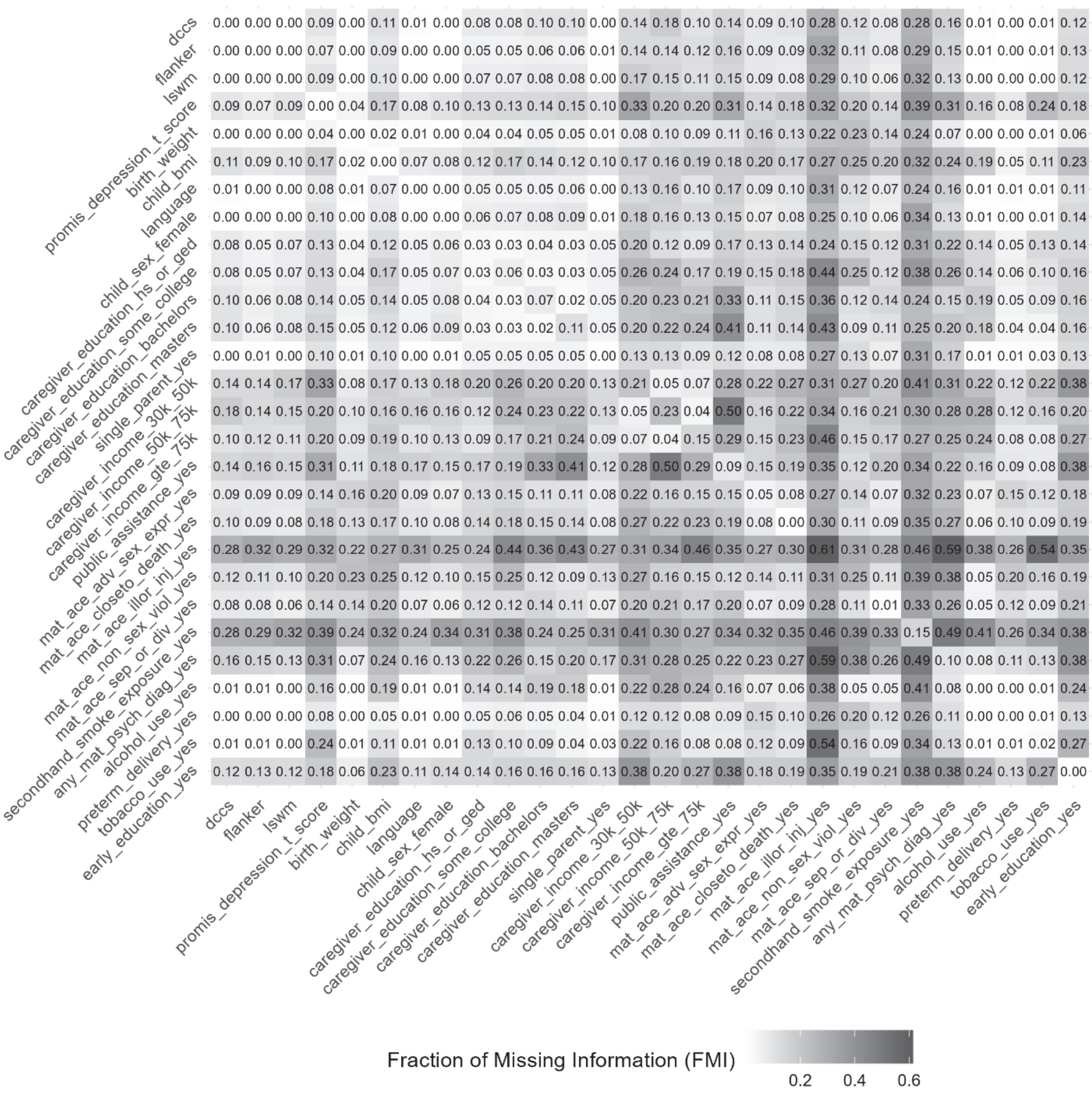

Note. DCCS = Dimensional Change Card Sort; LSWM = List Sort Working Memory; BMI = Body Mass Index; Mat = Maternal; Ace = Adverse Childhood Experiences; Adv = Adverse; Sex_Expr =

Sexual Experiences; Closeto_Death = Death of Someone Close; Illor_Inj = Illness or Injury; Non_Sex_Viol = Non-sexual Violence; Psych_Diag = Psychiatric Diagnosis; Sep_or_Div = Separation or Divorce.

Supplemental Table S2. Sample demographic information and cognitive outcomes compared across the six included cohorts.

**Characteristics**	PASS (*N* = 210)	CANDLE(*N* = 471)	ECHO-NOVI (*N* = 222)	VDAART (*N* = 183)	VCSIP (*N* = 45)	PRISM (*N* = 164)
**Child Sex**						
Female	124 (59%)	241 (51.2%)	109 (49.1%)	87 (47.5%)	24 (53.3%)	72 (43.9%)
Male	86 (41%)	230 (48.8%)	113 (50.9%)	96 (52.5%)	21 (46.7%)	92 (56.1%)
**Child Race**						
American Indian or Alaska Native	29 (13.8%)					
Asian		<5	15 (6.8%)	<10		5 (3%)
Black		306 (65%)	38 (17.1%)	98 (53.6%)	140 (22.2%)	36 (22%)
Native Hawaiian or other Pacific Islander			<5	<5		
Multiple Races	15 (7.1%)	32 (6.8%)	39 (17.6%)	20 (10.9%)	7 (15.6%)	15 (9.1%)
Other Race		<5	12 (5.4%)	<5		14 (8.5%)
White	166 (79%)	127 (27%)	106 (47.7%)	55 (30.1%)	28 (62.2%)	84 (51.2%)
Unknown		<5	<15	<5		10 (6.1%)
**Child Ethnicity**						
Hispanic	16 (7.6%)	<20	59 (26.6%)	56 (30.6%)	<10	61 (37.2%)
Non-Hispanic	194 (92.4%)	451 (95.8%)	163 (73.4%)	127 (69.4%)	38 (84.4%)	103 (62.8%)
Unknown		<5			<5	
**Caregiver Education**						
Less than High School	7 (3.3%)	24 (5.1%)	14 (6.3%)	30 (16.4%)	7 (15.6%)	26 (15.9%)
High School Degree	12 (5.7%)	175 (37.2%)	55 (24.8%)	49 (26.8%)	10 (22.2%)	10 (6.1%)
Some College	24 (11.4%)	60 (12.7%)	76 (34.2%)	64 (35%)	21 (46.7%)	38 (23.2%)
Bachelor's Degree	37 (17.6%)	117 (24.8%)	<55	21 (11.5%)	<5	<40
Master's Degree or Higher	20 (9.5%)	95 (20.2%)	25 (11.3%)	19 (10.4%)		49 (29.9%)
Unknown	110 (52.4%)		<5		<10	<5
**Household Income**						
< $30,000	54 (25.7%)	243 (51.6%)	39 (17.6%)	66 (36.1%)	5 (11.1%)	34 (20.7%)
$30,000 - $49,999	40 (19%)	70 (14.9%)	<40	24 (13.1%)	<5	<5
$50,000 - $74,999		92 (19.5%)	51 (23%)	21 (11.5%)	<5	<15
$75,000 or more		57 (12.1%)	91 (41%)	22 (12%)	<5	<55
Unknown	116 (55.2%)	9 (1.9%)	<5	50 (27.3%)	<30	67 (40.9%)
**Caregiver Relationship Status**						
Married or Cohabitating	176 (83.8%)	261 (55.4%)	177 (79.7%)	118 (64.5%)	15 (33.3%)	126 (76.8%)
Not Married or Cohabitating	34 (16.2%)	210 (44.6%)	<45	65 (35.5%)	<30	31 (18.9%)
Unknown			<5		<10	7 (4.3%)
**Early Childcare Education**						
Yes	41 (19.5%)	130 (27.6%)	205 (92.3%)	109 (59.6%)	6 (13.3%)	12 (7.3%)
No	17 (8.1%)	321 (68.2%)	<5	74 (40.4%)	<10	137 (83.5%)
Unknown	152 (72.4%)	20 (4.2%)	<20		<35	15 (9.1%)
**Public Assistance**						
Yes	<80	155 (32.9%)	165 (74.3%)	141 (77%)	39 (86.7%)	<5
No	<135	316 (67.1%)	57 (25.7%)	42 (23%)	<5	
Unknown	<5				<5	<165
**Family Psychiatric History**						
Yes	74 (35.2%)	26 (5.5%)	123 (55.4%)		39 (86.7%)	47 (28.7%)

No	136 (64.8%)	412 (87.5%)	<100		<5	<120
Unknown		33 (7%)	<5	183 (100%)	<5	<5
**Secondhand Smoke Exposure**						
Yes	14 (6.7%)	130 (27.6%)	29 (13.1%)	42 (23%)	38 (84.4%)	
No	84 (40%)	299 (63.5%)	138 (62.2%)	<5	<10	<5
Unknown	112 (53.3%)	42 (8.9%)	55 (24.8%)	<140	<5	<165
**Preterm Delivery**						
Yes	31 (14.8%)	33 (7%)	222 (100%)	15 (8.2%)	<5	<10
No	179 (85.2%)	438 (93%)		168 (91.8%)	<45	<160
Unknown						
**Prenatal Alcohol Use**						
Yes	134 (63.8%)	44 (9.3%)	<10	12 (6.6%)	<5	<105
No	76 (36.2%)	427 (90.7%)	213 (95.9%)	171 (93.4%)	34 (75.6%)	62 (37.8%)
Unknown			<5		<10	<5
**Prenatal Tobacco Use**						
Yes	44 (21%)	<40	<35	10 (5.5%)	36 (80%)	13 (7.9%)
No	166 (79%)	435 (92.4%)	189 (85.1%)	173 (94.5%)	<5	<155
Unknown		<5	<5		<10	<5
**DCCS**	98.00 (14.51)	92.90 (15.69)	90.41 (14.56)	93.77 (17.06)	96.07 (13.65)	94.41 (14.82)
**Flanker**	97.48 (11.67)	91.41 (14.67)	94.27 (14.27)	90.34 (14.64)	94.44 (12.62)	98.21 (13.24)
**LSWM**	98.12 (16.10)	96.07 (15.03)	90.13 (14.59)	96.69 (14.82)	93.18 (9.80)	99.90 (14.55)
**Language**	102.53 (14.34)	96.07 (16.02)	100.38 (15.44)	94.71 (14.56)	101.93 (12.65)	104.67 (15.93)
**Birthweight**	3402.89 (519.02)	3250.00 (531.59)	951.44 (276.77)	3263.08 (539.39)	2977.11 (502.87)	3338.27 (468.21)

Note. All cells with frequencies less than five are masked to protect the identity of our participants. We also masked all other cells which, in conjunction with the total cohort size, could potentially reveal the value of cells with frequencies less than five. DCCS = Dimensional Change Card Sort; LSWM = List Sort Working Memory.

References

1. Jorgensen, T. D., Pornprasertmanit, S., Schoemann, A. M., & Rosseel, Y. (2025). semTools: Useful tools for structural equation modeling (R package version 0.5-7). https://CRAN.R-project.org/package=semTools (accessed on 7 July 2026).
2. Wright, M.N.; Ziegler, A. Ranger: A fast implementation of random forests for high dimensional data in C++ and R. *J. Stat. Softw.* **2017**, *77*, 1–17. https://doi.org/10.18637/jss.v077.i01
3. Breiman, L. Random forests. Mach. Learn. 2001, 45, 5–32, https://doi.org/10.1023/A:1010933404324.
4. Rodgers, D.M.; Jacobucci, R.; Grimm, K.J. A Multiple Imputation Approach for Handling Missing Data in Classification and Regression Trees. *J. Behav. Data Sci.* **2021**, *1*, 127–153, https://doi.org/10.35566/jbds/v1n1/p6.
5. Enders, C. K. (2010). Applied missing data analysis. Guilford Press.
6. Campion, W.M.; Rubin, D.B. Multiple Imputation for Nonresponse in Surveys. *J. Mark. Res.* **1989**, *26*, 485, https://doi.org/10.2307/3172772.
